# Feasibility of using an led-probe in third-space endoscopy: a clinical study

**DOI:** 10.1186/s12876-020-01260-9

**Published:** 2020-05-05

**Authors:** Oscar Víctor Hernández Mondragón, Raúl Zamarripa Mottú, Omar Solórzano Pineda, Raúl Alberto Gutierrez Aguilar, Luís Fernando García Contreras

**Affiliations:** Division of Endoscopy, Specialties Hospital, National Medical Center Century XXI, Cuauhtémoc Avenue 330, 06700 México City, Mexico

**Keywords:** Third-space endoscopy, LED-probe, Peroral endoscopic myotomy, Gastric peroral endoscopic myotomy

## Abstract

**Background:**

Third-space endoscopy is a novel, safe, and effective method for treating different gastrointestinal conditions. However, several failed endoscopic procedures are attributed to incomplete myotomy. Lighting devices are used to prevent organic injuries. We aimed to investigate the feasibility of using a hand-made LED-probe (LP) in third-space procedures.

**Methods:**

This prospective study was conducted in a tertiary-care center in Mexico between December 2016 and January 2019. We included peroral endoscopic myotomy (POEM) and gastric peroral endoscopic myotomy(G-POEM) procedures. Pseudoachalasia, peptic ulcer, normal gastric emptying scintigraphy (GES) and prepyloric tumors were excluded. LP was used to guide or confirm procedures. Clinical and procedural characteristics were recorded and analyzed.

**Results:**

Seventy third-space procedures were included (42POEM,28G-POEM), with an average patient age of 46.7 ± 14.3 and 43.7 ± 10.1 years, respectively. For the POEM and G-POEM groups, respectively, 18/42(42.9%) and 13/28(46.7%) patients were males; median procedure times were 50 (interquartile range [IQR]: 38–71) and 60(IQR: 48–77) min, median LP placement times were 5(IQR: 4-6) and 6(IQR: 5-7) min, mild adverse events occurred in 4(9.4%) and 4(14.2%) of cases, and clinical success at 6 months occurred in 100 and 85.7% of cases. Integrated relaxation pressure (IRP) improved from 27.3 ± 10.8 to 9.5 ± 4.1 mmHg (*p* < 0.001); retention percentage at 4 h also improved. LP was successfully placed and adequate myotomy confirmed including 14.2 and 17.8% of POEM and G-POEM difficult patients.

**Conclusions:**

Using an LP is promising and allows guiding during third-space procedures either for submucosal tunnel creation or myotomy confirmation, with excellent safety and efficacy in clinical practice.

## Background

Third-space endoscopy has emerged as a novel and effective method for the treatment of different gastrointestinal disorders such as achalasia and refractory gastroparesis [1–4]. However, difficult or incomplete myotomy has been identified in peroral endoscopy myotomy (POEM) and gastric-peroral endoscopy myotomy (G-POEM) as a result of different factors such as end-stage disease or previously treated cases (due to high submucosal fibrosis) [1, 5], inability to identify the esophagogastric junction (EGJ) throughout the submucosal tunnel during POEM [6–8] or the pyloric muscle ring (PMR) during G-POEM [9], lack of third-space endoscopy experience [2, 3, 6], and certain anatomical factors (megaesophagus, sigmoid-type) [4, 9, 10]. These factors are associated with a failed procedure. Different techniques, such as fluoroscopy [9, 11] or double endoscopy [12, 13] have been proposed to overcome these problems and guide an appropriate tunnel creation and myotomy; however, low availability and high costs limit their use.

Lighting devices for surgical guidance have been investigated for years with good results. In laparoscopic colorectal and gynecological surgeries, the use of lighted ureteral stents has successfully prevented iatrogenic injuries [14, 15]. In ophthalmologic procedures, light-emitting diode (LED) technology is used for vitrectomy, with the advantage of no thermal or photodynamical harm; this provides better illumination than other light sources [16, 17]. In third-space endoscopy, the use of a dedicated LED-probe (LP) that could be inserted into the submucosal tunnel (cornerstone of the procedure) [1, 2, 8] could be used to guide the direction during the procedure or confirm a correct myotomy when combined with the endoscope, before closing the entry site. Use of an LP could be an excellent alternative during third-space endoscopic procedures. The aim of this study was to evaluate the feasibility of using of LP in clinical practice, especially during POEM or G-POEM third-space procedures.

## Methods

### Study design and ethical considerations

This prospective study was conducted in a tertiary care center in Mexico City, Mexico between December, 2016 and January, 2019. We included patients between the ages of 18 and 90 years with naïve or previously-treated achalasia or severe refractory gastroparesis and who were treated with POEM or G-POEM, respectively, with 6 months of follow-up. Patients with pseudoachalasia were excluded from the POEM procedure. Active peptic ulcer disease, normal gastric emptying scintigraphy (GES) and prepyloric tumor lesions were excluded from G-POEM. Patients with severe clinical conditions that could be contraindications to any of these procedures were also excluded (severe chronic obstructive pulmonary disease, recent myocardial infarction). This protocol was approved by the Local Ethics Committee (R-2016-3601-192; registration number: 2016-CMN675). Informed consent was obtained from all patients.

### Poem

#### Patients

The diagnosis of achalasia was based on the Chicago Classification [18], using high resolution manometry (HRM). Upper gastrointestinal (GI) endoscopy, computed tomography (CT) scanning, a timed barium esophagogram (TBE) and the Chagas disease test were performed. Esophageal classification was evaluated according to Rezende’s classification [19]; the Eckardt score was used for clinical evaluation [20].

#### Procedure

The esophagus was cleaned 24 h before the POEM procedure and antibiotic prophylaxis with third-generation cephalosporines or quinolones was administered. All procedures were performed under general anesthesia. A 9. 8-mm outer diameter with a 2. 8-mm working channel endoscope was used (EG590WR; Fujinon, Tokyo, Japan), along with a transparent cap model (DH-28GR, Fujinon). An electrosurgical unit (ERBE VIO-200D, Tübingen, Germany), and an I-type hybrid knife (ERBE, Tübingen, Germany) were also used. Closure was performed using hemoclips (Boston Scientific, Natick, Massachusetts, USA).

The LP system was created using a sterile polyurethane non-weighted 127 cm × 20 Fr nasogastric feeding tube (AMA Proveduria, Mexico City, Mexico) that was cut at the middle. A thin strip of 150cms long of an ultrabright slim LED strip light system of 1/8 in [3.5 mm] in diameter, with a capability of 112 lm/m (FlexfireLEDs, Costa Mesa, California, USA), was inserted throughout this catheter and attached to it with one 127 cm long strip of Scotch black tape (3 M, St. Paul, Minnesota, USA). This LP was coupled to a power supply system of 12 V that used conventional alkaline AA batteries (Duracell, Bethel, Connecticut, USA). After LP creation, it was cleaned with soapy water, rinsed and then dried. Finally, we used an antiseptic wipe (SoluPrep™, 3 M, St. Paul, Minnesota, USA), which has 2% w/v chlorhexidine gluconate and 70% v/v isopropyl alcohol, in order to clean the catheter before use (video 1).


**Additional file 1: Video 1.** Led Probe Construction. This is a video that we named led probe construction. In this video we show how to perform a LP before using in POEM or G-POEM cases.


**Fig. 1:**
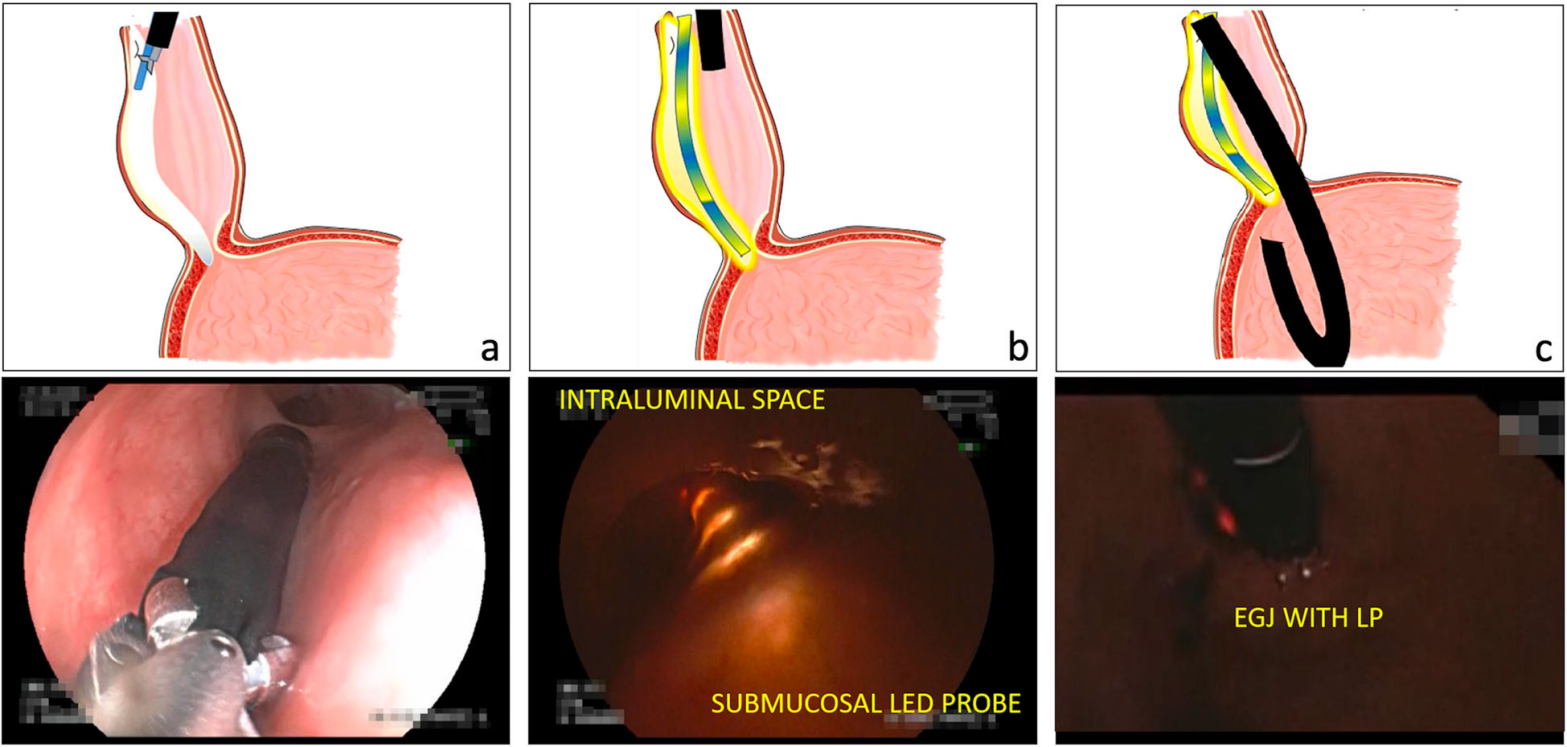
Animation and endoscopic image of POEM procedure guided and confirmed with LP. **a** LP is inserted into the submucosal tunnel. **b** Intraluminal view of LP while switched on **c** Endoscopic retroflex view at esophagogastric junction for confirmation of myotomy before entry site closure. LP = Led-probe, EGJ = Esophagogastric junction

The POEM technique was based on Inoue’s technique [21]. The technique was performed using the following procedure: (1), initially, the esophagus was cleaned if necessary; (2), injection and incision were performed with a mixture of saline solution and 0.5% methylene blue being injected 13-15 cm above the EGJ (20 cm for type III patients) and a 12-15 mm longitudinal incision being made for anterior (naïve patients) or posterior (previously-treated patients) approaches; (3), the submucosal tunnel was created, with the tunnel being created up to 3 cm below the EGJ; (4), a full-thickness myotomy was performed in all patients; (5), the LP was placed through the mouth; once it passed the cricopharyngeal muscle, it was grasped using biopsy forceps under endoscopic visualization and inserted up to the end of the submucosal tunnel; (6), intraluminal revision of the POEM procedure was performed and EGJ was reviewed in retroflexion with the LP on, in order to confirm that submucosal tunnel reached gastric space and an adequate myotomy was performed. If LP on was not observed, submucosal tunnel and myotomy were considered as insufficient and they were corrected as necessary until LP on was observed. Submucosal tunnel was reviewed again to rule out adverse events secondary to LP or endoscope at this level; (7), closure with clips at the entry site was performed **(**Fig. 1**).**

#### Follow-up

Twenty-four hours after POEM, upper GI endoscopy and upper GI series were performed to rule out adverse events. Antibiotics were continued by intravenous and then oral route for 7 days. A proton pump inhibitor (PPI) was administered for 4 weeks. Diet progressed from a liquid diet up to a normal one over the following 3 weeks. Follow-up continued for 6 months, with HRM, GI endoscopy, pHmetry, TBE, reflux questionnaires, and Eckardt scores evaluated at 3 and 6 months. Success was defined as an integrated relaxation pressure (IRP) < 15 mmHg, Eckardt score < 3, and TBE demonstrating adequate passage of contrast (≥ 80% at 5 min) 3 and 6 months postoperatively.

### G-poem

#### Patients

Gastroparesis was diagnosed based on clinical evaluations and scintigraphy. Severe refractory gastroparesis was based on the presence of delayed gastric emptying-related symptoms, including nausea, retching, vomiting, abdominal pain, post-prandial fullness, early satiety and/or bloating. Patients who had failure or recurrence after receiving optimal pharmacological therapies and a Gastroparesis Cardinal Symptom Index (GCSI) score > 2.3 (score that has been validated as severe when greater than 2.3) [22] with a retention percentage at 4 h (RP4H) in GES > 10% and a mean half emptying time (MHET) > 150 min, were also diagnosed with severe refractory gastroparesis. Efficacy was evaluated based on a reduction in the self-reported gastroparetic symptoms of the patients; the absence of recurrent hospitalizations and the proportion of patients with a decrease in GCSI score < 2.3, RP4H < 10% and MHET< 150 min. Adverse events in POEM and G-POEM were graded according to the American Society for Gastrointestinal Endoscopy Lexicon [23].

#### Procedure

Procedures were performed in the endoscopic unit under general anesthesia. Forty-eight hours before the procedure, all patients were administered a liquid diet and antibiotic prophylaxis with third-generation cephalosporines or quinolones. Endoscopic instruments were the same as in POEM patients, including the LP.

**Fig. 2:**
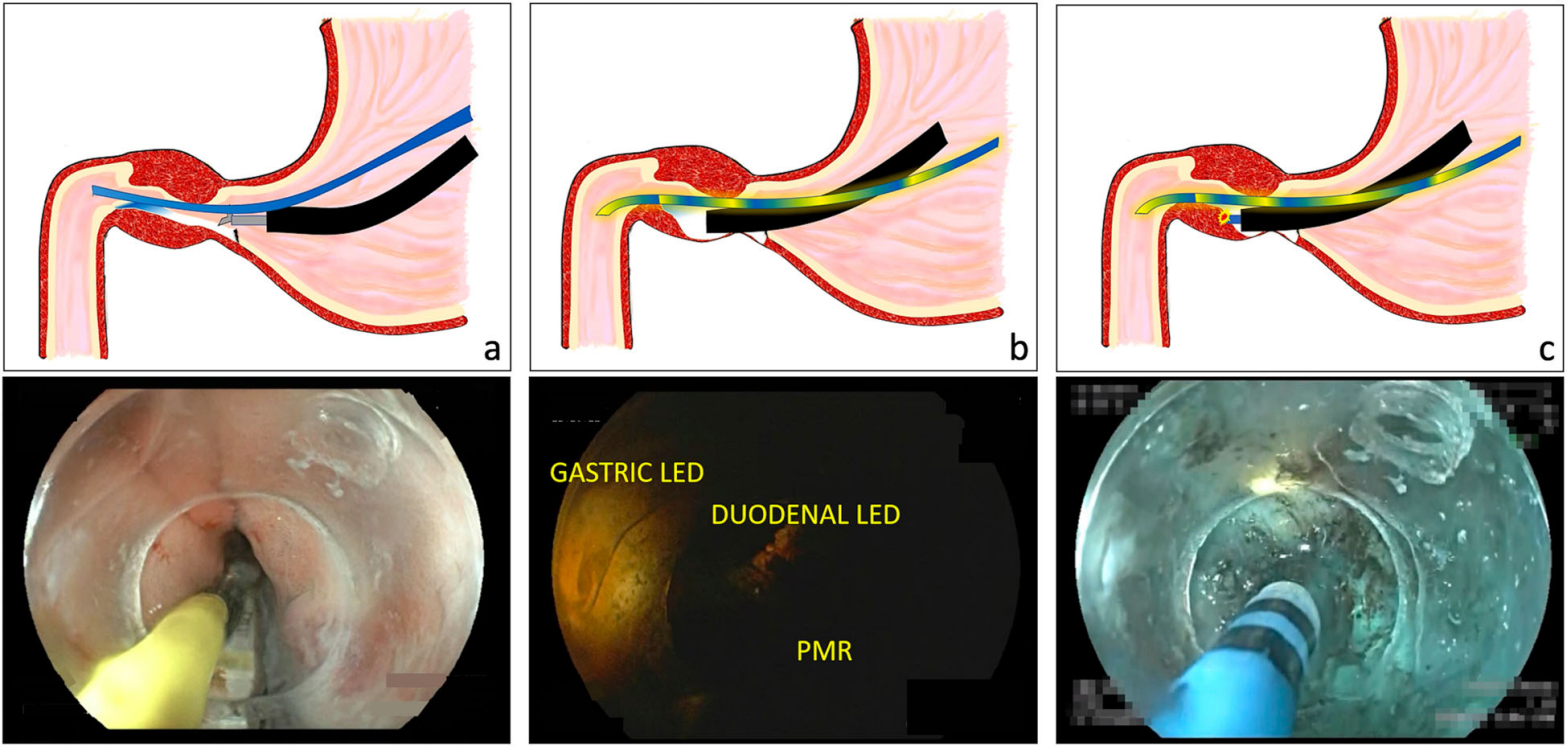
Animation and endoscopic image of G-POEM procedure guided and confirmed with LP. **a** LP is inserted intraluminally into the duodenal bulb. **b** Endoscopic view of LP throughout the submucosal space. Gastric and duodenal LED lights are observed with PMR at the bottom. **c** Myotomy is performed with LP guidance

The G-POEM procedure was based on Khashab’s technique [24]. The technique was performed based on the following protocol: (1), revision and cleaning of the stomach were performed; (2), injection and incision were performed 5 cm before the pylorus for submucosal bleb creation using a combination of saline solution with 0.5% methylene blue. A longitudinal 1.5 cm incision was also performed; (3), a submucosal tunnel was performed in pyloric direction until PMR was identified. If submucosal tunnel orientation was lost during this step, LP was used and was inserted throughout the mouth, grasped with biopsy forceps and placed intraluminally into the duodenal bulb in order to guide submucosal tunnel creation and PMR identification; (4), myotomy of the circular and longitudinal muscular layers of the pylorus and the antrum was performed; the incision was 3-4 cm in length and deep up to the serosa. LP was used for myotomy confirmation in all cases. Before closure was performed, submucosal tunnel was reviewed endoscopically to rule out complications at this level; (5), the incision was closed using the over the scope clips (OTSC) type A (OVESCO Endoscopy, AG, Tübingen, Germany) or hemoclips **(**Fig. 2**).**

#### Follow-up

Patients were admitted to the hospital after the procedure and given intravenous antibiotics, and then changed to oral route, in order to complete 7 days. An upper GI series and upper endoscopy were performed at 24 h to rule out adverse events. Diet progressed from a liquid diet to a normal diet over the next week. PPI was administered for 4 weeks. Both GCSI and GES were performed 3 and 6 months postoperatively.

### Statistical analyses

Sample size was calculated based on an assumption that there would be at least 90% of efficacy in the completion of third-space procedures when LP is used, with a significance level of 0.05 (type I error of 5%) and a beta of 0.20 (type II error of 20%). Using an online statistically-validated program for sample size calculation (EpiInfo, USA), we calculated a minimum of 25 patients for POEM and G-POEM procedures. Quantitative data were expressed as mean (standard deviation [SD]) or median with interquartile range (IQR); qualitative data were expressed as frequencies and percentages. Bivariate comparisons were done using the Friedman test, chi-squared test and a one-way analysis of variance, as appropriate. *P* < 0.05 was considered to be statistically significant. SPSS v.23.0 (IBM, Chicago, Illinois, USA) was used for all statistical analyses.

## Results

There were 70 third-space procedures performed between December 2016 and January 2019 (42 POEM and 28 G-POEM).

### Poem

#### Baseline characteristics

Forty-two patients were included with a mean age of 46.7 ± 14.3 years and 18 (42.9%) were male. Esophagus type grade II (16 [38%]) and achalasia subtype II (20 [47.6%]) were the most common. Thirty-two (76.2%) were naïve, while 10 (23.8%) had been previously treated, (60% had undergone LHM, 20% botulinum toxin injection, and 20% pneumatic dilation). No Chagas disease was found.

#### Procedure

The median total POEM time was 50 min (IQR 38–71). The mean tunnel and myotomy lengths were 12.9 ± 3.6 cm and 10.5 ± 3.1 cm, respectively. The most common mild adverse event was minor bleeding in 2 cases (4.7%), and the median length of stay (LOS) was 3 days (IQR 1-4).

#### Efficacy

Clinical response was observed in all cases at the 6-month follow-up. The Eckardt score decreased from 9 to 1 at the 3-month follow-up (*P* < 0.001) and did not change at the 6-month follow-up (*P* < 0.001). The IRP decreased from 27.3 ± 10.8 mmHg to 9.8 ± 3.8 mmHg at the 3-month follow-up (*P* < 0.001) and 9.5 ± 4.1 mmHg at the 6-month follow-up (*P* < 0.001); TBE showed emptying of < 50% in 100% of patients to emptying of > 50% in 100% (*p* > 0.001) after 6 months. Furthermore, 57% presented positive pHmetry, 15% had esophagitis and 12% had clinical symptoms of reflux disease (Table 1).

**Table 1:** Characteristics of the 42 POEM procedures performed with LED probe

Patients *N* = 42	Value			
Age, mean (SD), years	46.7 ± 14.3			
Sex,male,n (%)	18 (42.9%)			
Type of esophagus, n (%)				
• Normal	2 (5%)			
• Grade I	8 (19%)			
• Grade II	16 (38%)			
• Grade III	8 (19%)			
• Grade IV	8 (19%)			
Previous treatments, n (%)				
• *Treatment naïve*	32 (76.2%)			
• *‘Previously treated*	10 (23.8%)			
• +Post-LHM	6 (60%)			
• +Botulinum toxin injection	2 (20%)			
• +Pneumatic dilation	2 (20%)			
Achalasia subtype, n (%)				
• Type I	11 (26.2%)			
• Type II	20 (47.6%)			
• Type III	11 (26.2%)			
**Procedure**				
Tunnel length, mean (SD), cm	12.9 ± 3.6			
Myotomy length, mean (SD) cm	10.5 ± 3.1			
LP placement time, median (IQR), min	5 (4–6)			
Patients with inadequate myotomy after initial classic POEM that benefited from LP use (difficult cases), n (%)	6 (14.2%)			
Total POEM time, median (IQR), min	50 (38–71)			
Adverse Events, n (%)	4 (9.4%)			
• Minor bleeding	2 (4.7%)			
• Pneumoperitoneum	2 (4.7%)			
**POEM outcomes**	**PRE-POEM**	**POST-POEM 3 m**	**POST-POEM 6 m**	***P*value**
Eckardt score, median (IQR), points	9 (6–12)	1 (0–3)	1 (0–3)	< 0.001^1^
IRP pressure, mean (SD), mmHg	27.3 ± 10.8	9.8 ± 3.8	9.5 ± 4.1	< 0.001^2^
TBE				< 0.001^3^
• < 50%	100%	0%	0%	
• 50–80%	0%	14%	9.5%	
• > 80%	0%	86%	90.5%	

*SD* standard deviation, *IQR* interquartile range, *POEM* peroral endoscopic myotomy, *LP* led-probe, *LHM* laparoscopic Heller myotomy

^1^ Friedman test

^2^ ANOVA test

^3^ X^2^ test

### G-poem

#### Baseline characteristics

Twenty-eight patients with a mean age of 43.7 ± 10.1 years were included and 13 were male (46.4%). The most common etiology was diabetes in 12 (42.9%) and the mean duration of disease before G-POEM was 22.2 ± 5.5 months. The most predominant symptoms and previous therapy were: nausea and vomiting in 15 (53.5%), and medical therapy in 22 (78.7%), respectively. The median number of hospitalizations preoperatively was 2 (IQR 2-5).

#### Procedure

The median total G-POEM time was 60 min (IQR 48–77). The mean tunnel and myotomy lengths were 5.2 ± 0.96 cm and 3.2 ± 0.82 cm, respectively. The most common mild adverse event was capnoperitoneum in 2 patients (7.1%); it required abdominal decompression with a Veress needle and 1 mucosal tear 24 h after procedure and was solve endoscopically with clips. The median LOS was 2 days (IQR 1-6).

#### Efficacy

The GSCI score decreased from 3.5 ± 0.64 points to 1.8 ± 0.61 after 3 months (*P* < 0.001), and 1.2 ± 0.43 after 6 months (*P* < 0.001). GES test showed a decrease in RP4H from 35.3 ± 11.6 to 11.1 ± 4.2 after 3 months (*P* < 0.001), and 9.3 ± 3.2 after 6 months (*P* < 0.001). The half-emptying time improved from 260.2 ± 66.9 min to 165.9 ± 31.2 min after 3 months (*P* < 0.001), and 152.7 ± 23.1 min after 6 months (*P* < 0.001). Clinical response was observed in 24 patients (85.7%) at the 6-month evaluation. Resolution of the predominant symptoms were as follows: resolved in 16 (57.1%), 18 (64.3%) and 9 (32.1%); improved in 10 (35.8%), 5 (17.8%) and 11 (39.2%); not changed in 1 (3.6%), 3 (17.8%) and 7 (25%) and worsened in 1 (3.6%), 2 (7.2%) and 1 (3.6%), for nausea/vomiting, abdominal pain and gastric fullness, respectively. GES was normalized in 17 (60.7%) and partially improved in 8 (28.5%) of patients at the 6-month evaluation (Table 2).

**Table 2:** Characteristics of the 28 G-POEM procedures performed with LED probe

Patients *N* = 28	Value			
Age, mean (SD), years	43.7 ± 10.1			
Sex, male, n (%)	13 (46.4)			
Etiology, n (%)				
• Diabetic	12 (42.9%)			
• Idiopathic	11 (39.2%)			
• Postsurgical	5 (17.9%)			
Duration of disease before G-POEM, mean (SD), months	22.2 ± 5.5			
Predominant symptoms, n (%)				
• Nausea/vomiting	15 (53.5%)			
• Abdominal pain	8 (28.6%)			
• Gastric fullness	5 (17.9%)			
Previous therapy, n (%)				
• Medical treatment	22 (78.7%)			
• Botulinum toxin injection	5 (17.8%)			
• Transpyloric stenting	1 (3.5%)			
**Procedure**				
Tunnel length, mean (SD), cm	5.2 ± 0.96			
Myotomy length, mean (SD), cm	3.2 ± 0.82			
LP placement time, median (IQR), min	6 (5-7)			
Patients with inadequate submucosal tunnel direction after initial classic G-POEM procedure that benefited from LP use, n (%)	5 (17.8%)			
Total G-POEM time, median (IQR), min	60 (48–77)			
Adverse Events, n (%)	4 (14.2%)			
• Capnoperitoneum	2 (7.1%)			
• Mucosal tear	1 (3.5%)			
• Prepyloric ulcer	1 (3.5%)			
**G-POEM outcomes**	**PRE-GPOEM**	**POST-GPOEM 3 m**	**POST-GPOEM 6 m**	***P*value**
GSCI score, mean (SD), points	3.5 ± 0.64	1.8 ± 0.61	1.2 ± 0.43	< 0.001^1^
RP4H, mean (SD), percentage	35.3 ± 11.6	11.1 ± 4.2	9.3 ± 3.2	< 0.001^1^
MHET, mean (SD), minutes	260.2 ± 66.9	165.9 ± 31.2	152.7 ± 23.1	< 0.001^1^

*SD* standard deviation, *IQR* interquartile range, *G-POEM* gastric peroral endoscopic myotomy, *LP* led-probe, *GSCI* gastroparesis cardinal symptoms index, *RPH*4 retention percentage 4 h, *MHET* mean half emptying time

^1^ ANOVA test

### LED probe

The median placement time for POEM and G-POEM were 5 (IQR 4-6) and 6 (IQR 5-7) min, respectively. All probes were successfully placed without adverse events and no adverse events or technical failures were observed after procedures secondary to their use. There was no damage to the submucosal space or at the intraluminal mucosal level in both POEM and G-POEM cases, and we didn’t have any associated infections, neither when LP was placed in the mediastinal space at EGJ level in POEM cases. In general, LP use helped to adequately complete POEM and G-POEM in 11/70 (15.7%) of cases. After initial classic POEM procedure, LP on was placed and not observed in 6/42(14.2%) of cases. Therefore, submucosal tunnel and myotomy were extended up to an adequate LP confirmation. In POEM cases, placement of LP in posterior approach was relatively easier than anterior approach, but without significant differences in placement times (4 vs 6 min; *P* = 0.2). In G-POEM cases, inadequate submucosal direction was found in 5/28(17.8%) of cases and LP use helped to correct it and confirm myotomy of the PMR in all cases. (video 2).


**Additional file 2: Video 2.** LP clinical cases. In this video we show the use of LP in clinical POEM and G-POEM cases.


## Discussion

In this study we evaluated the feasibility of using a new device, the LP, and confirmed its safe and effective use in clinical practice when performing third-space procedures (POEM and G-POEM).

Third-space endoscopy was first described by Sumiyama et al. [25]; it was first used in animals in 2007, and then used in humans. It is based on the creation of a submucosal tunnel to perform surgical procedures with confirmed safety and efficacy. It transforms the concept of endoluminal endoscopy to intramural, making many diseases that previously would have been treated by open or laparoscopic surgery endoscopically curable [1, 4].

The lumen is considered as the first space, while the peritoneum is considered the second space and the intentionally-created tunnel is the third space (space between the mucosa and muscularis propria) [2–4]. Different disorders have been addressed by this technique, including Zenker’s diverticulum, myotomy for achalasia, gastroparesis, Hirschsprung’s disease, removal of tumors arising from the muscularis propria and beyond, and stricture treatment [1, 2, 8]. Among them, achalasia and gastroparesis are the most prevalent diseases treated by this technique. However, “difficult”, “incomplete”, or even “not possible” tunnel creation or myotomy have been described and are associated with certain complexity factors such as end-stage disease (POEM) [5], previously-treated cases (POEM and G-POEM) [4, 8, 24], inability to identify EGJ (POEM) [6–8], PMR (G-POEM) [9], high submucosal fibrosis (POEM and G-POEM) [2–4, 6–8], lack of experience (POEM and G-POEM) [2, 3, 6],or anatomical factors (POEM) [1, 4, 9, 10]. These factors result in a failed procedure, even if the endoscopist believes that the procedure was successful [1, 3]. If the tunnel is too short, the procedure is ineffective; if the length of the myotomy is too long, there is a higher risk of adverse events, including perforation or bleeding [3, 12]. Currently, there are several endoscopic landmarks, such as palisading vessels at the EGJ and the circular bundle of LES fibers in POEM (difficult use and inaccurate), or the continuous insertion and extraction of the endoscope from the tunnel, that are used to identify the PMR, which is technically challenging. A second endoscope (POEM and G-POEM) [12, 13] and fluoroscopy (G-POEM) [9, 11] are used in an attempt to overcome these problems; however, these methods are costly or are unavailable. Therefore, we decided to explore a new alternative to overcome these problems when performing third-space endoscopy.

The use of lighting devices has been explored in medicine for years. In colorectal and gynecological surgeries, for example, iatrogenic ureteric injury is a serious complication with a variable incidence between 0. 7-10% [14]. The identification of ureters is challenging and the optional double J stent placement is invasive and associated with serious adverse events [15];however, the use of fluorescence and lighted ureteral stents has overcome these problems. LED technology was invented in 1907 by H. J. Round but was commercially available in 1962 in electrical components. Modern LED technology with more practicality was used after 2010 [17]; uses in medicine are confirmed in ophthalmologic procedures where improved illumination for vitrectomy has been observed [16].LED has advantages over incandescent light sources. It provides lower energy consumption, a longer lifetime, smaller size, faster switching, a better spectrum of light and intensity (emitting more lumens per watt compared with light bulbs), and cool light that radiates minimal heat. It is safe because mercury or other hazardous metals are not contained within it [16, 17].

Because of safety and efficiency, we decided to use a white LED-probe and orally insert it into a conventional 127 cm × 20 Fr nasogastric feeding tube. We spent between 10 to 15 min for LP building and disinfection process. Excellent visualization was obtained with the 112 lm/m of the probe; this was enough to be visualized under the submucosal tunnel or over the intraluminal space when inserted into the tunnel. We didn’t have technical problems during assembly or during procedures, neither when insertion into the patient was performed or after LP was used and procedures were finished. Therefore, based on these results, we confirmed the safety and efficacy of this device, that had a median insertion time of 5 min (4-6) for POEM and 6 (5-7) for G-POEM, without compromising total procedural times, and being similar to those observed in previous studies [5–7, 10, 11, 21, 24].

POEM was performed as described by other groups [5, 6, 21], with similar demographic characteristics. However, in our cohort 23.8% were previously treated and 38% grade III and IV. These are the subgroups theoretically more difficult to treat; therefore, with the greatest benefit if LP is used. Nonetheless, POEM was completed in 100% of cases and the mean myotomy length was 10.5 ± 3.1 cm, which is similar to the length of 9.4 ± 3.1 cm obtained in other studies [6]. Grimes et al. [13] compared double-scope vs conventional POEM in a clinical trial that included 50 patients per group. No differences in technical (98% vs 100%) or clinical success (93% vs 97%) was found, but with a 34% longer myotomy and 17 min increase in procedural times for double-scope group. In our study, the LP was 6 mm in diameter, which is similar to the length of the neonatal endoscope used in Grimes’ study. However, our LP system was advantageous in terms of cost (10 dls per LP), and placement time (5 min vs 17 min). We confirmed an adequate myotomy in all cases including 6 patients (14.2%) who were considered as difficult (4 grade IV, 1 grade III and 1 pneumatic dilation), in whom classic POEM didn’t complete myotomy and who benefited from the LP use, avoiding potential adverse events or risk of incomplete procedure. Additionally, no other endoscopy tower was needed (saving costs and space in the endoscopy room).. In 2019, Grimes et al. published the follow-up of the cohort of double-scope vs conventional POEM, with a median of 3 years. They found no differences in clinical outcomes between groups (83% vs 80%; *P* = 1.0), without differences in reflux disease incidence, but more cases with grade B esophagitis were presented in treatment group (25% vs 4%; *P* = 0.049); they hypothesize that this is because a longer myotomy is performed in them (1.6 ± 1.2cms) [26]. In our cases, the LP allowed performing an adequate EGJ myotomy and, at 6-month evaluation, clinical outcomes and the occurrence of reflux disease was similar to those of other studies [5–8, 12, 13, 21]. This suggests that the adequate confirmation of EGJ myotomy is the most important step in POEM procedures, regardless of whether an external device is used or not. Confirmation, which should be performed in all cases, mostly in early-experienced endoscopists in POEM procedure, represents LP as an excellent alternative for this purpose.

We performed the G-POEM and LP placement in all cases. Demographic characteristics were similar to other groups [8–11, 24]. The pylorus was previously manipulated in 21.4% of cases (botulinum toxin injection and transpyloric stent), potentially difficult cases. However, median G-POEM time (60 min) was similar to other groups [1, 9–12, 24]. Xue H et al. [9] compared the use of fluoroscopy-guided G-POEM vs conventional G-POEM procedure in 14 patients; all procedures achieved technical success, the PMR was identified in all 7 patients of the fluoroscopy-guided group, and only in 4(57.1%) from the control (*P* < 0.03). However, this was not clinically expressed, with a non-statistically significant difference between GCSI and GES. In our group, LP provided a better orientation towards PMR identification and myotomy confirmation in all cases, including 5/28 patients where the endoscopist was “lost”, during tunnel creation and PMR was not identified, where the LP allowed the completion of G-POEM procedures, representing a 17.8% benefit in them. However, besides the fact that the G-POEM outcomes were slightly better in our study, when compared with other centers, with a general clinical success (85.7% vs 69–81%) in GCSI and GES (89.2% vs 69–84.2%) at 6-month evaluations, we can’t assume that this could be explained because of the 100% PMR identification (similar to the fluoroscopy-guided group from Xue’s study). However, as stated by other authors, different gastroparesis subtypes with their corresponding physiopathology could explain the real heterogeneous mid-term results more than the simple direct effect of the PMR cutting, inclusive with LP guidance, as in our patients [1–4, 9–11, 24].

The strengths of our study include the use of LP in the two most common and important third-space procedures, the sample size that was reached in both and calculated for statistical significance, adequate and strict procedural and follow-up protocols, technical confirmation of all steps in all cases, and excellent safety without adverse events associated with LP use. Our study also has limitations that should be addressed. First, LP is not yet commercially available. Second, LP has to be made before each procedure by the medical doctor, which, in spite of the fact that it takes only between 10 and 15 min, could be time-consuming. Third, different LED and nasogastric feeding tube brands exist around the world, which limits the availability of the system we used. Fourth, only POEM and G-POEM cases were included; pediatric and other third-space procedures were not included, and fifth, LP was used in 76.2% of naïve POEM cases, which represent a subgroup of non-difficult cases in which LP could have been useless, especially when performed by highly-experienced endoscopists in third-space procedures; therefore, we think that the best advantage of LP use could be found in early-experience endoscopists in third-space procedures. However, we think that LP is a useful device for POEM and G-POEM procedures because of its simplicity, innovation, low costs, safety and the ability to make difficult procedures potentially easier.

## Conclusion

In conclusion, we have confirmed the feasibility of using LP in third-space endoscopy as a new alternative to performing POEM or G-POEM cases, being specifically useful when classic anatomical landmarks are not completely reliable, in difficult cases, low-volume POEM and G-POEM centers, limited third-space procedures experience and when no other confirming methods are available. However, the real clinical relevance of LP use must be confirmed with longer evaluations and comparative studies. Commercialization and evaluation in other third-space procedures are necessary to elucidate potential advantages of the LP system.

## Supplementary information

**Additional file 3.** POEM files. This is the POEM files from our cohort of patients with POEM that underwent to LP procedure.

**Additional file 4.** G-POEM files. This is the G-POEM files from our cohort of patients with G-POEM that underwent to LP procedure.

## Data Availability

All data generated or analyzed during this study are included in this published article [we added spv files as supplementary files].

## Abbreviations

LP: LED-probe
POEM: Peroral endoscopic myotomy
G-POEM: Gastric peroral endoscopic myotomy
GES: gastric emptying scintigraphy
IQR: interquartile range
IRP: Integrated relaxation pressure
EGJ: Esophagogastric junction
PMR: pyloric muscle ring
LED: Light-emitting diode
HRM: High resolution manometry
Upper GI: Upper gastrointestinal
CT: Computed tomography
TBE: Timed barium esophagogram
PPI: Proton pump inhibitor
GCSI: Gastroparesis Cardinal Symptom Index
RP4H: Retention Percentage 4 h
OTSC: Over the scope clip
SD: Standard deviation
